# Association of Selected miRNAs (hsa-miR-27b, hsa-miR-128-3p, hsa-miR-145-5p, hsa-miR-552-3p) with HER2 Status and Chromosome 17 Centromere Copy Number Increase in Gastric Cancer

**DOI:** 10.3390/ijms27125184

**Published:** 2026-06-08

**Authors:** Maciej Ciesielski, Marzena Anna Lewandowska, Mariusz Szajewski, Krzysztof Pastuszak, Aleksandra Ciarka, Piotr Kurek, Jakub Walczak, Michał Stańczak, Jacek Zieliński, Wiesław Janusz Kruszewski

**Affiliations:** 1 Department of Oncological Surgery, Gdynia Oncology Centre, 81-519 Gdynia, Poland; mariusz.szajewski@gumed.edu.pl (M.S.); piotr_kurek@gumed.edu.pl (P.K.); jwalczak@szpitalepomorskie.eu (J.W.); mstanczak@gumed.edu.pl (M.S.); wieslaw.kruszewski@gumed.edu.pl (W.J.K.); 2 Department of Oncological Surgery, Faculty of Health Sciences with the Institute of Maritime and Tropical Medicine, Medical University of Gdańsk, 81-519 Gdynia, Poland; 3 Molecular Oncology and Genetics Department, Innovative Medical Forum, Łukaszczyk Oncology Centre, 85-796 Bydgoszcz, Poland; lewandowskam@co.bydgoszcz.pl; 4 Department of Thoracic Surgery and Tumors, Ludwik Rydygier Collegium Medicum in Bydgoszcz, Nicolaus Copernicus University, 85-067 Bydgoszcz, Poland; 5 Department of Algorithms and System Modelling, Gdańsk University of Technology, 80-233 Gdańsk, Poland; krzpastu@pg.edu.pl; 6 Laboratory of Translational Oncology, Intercollegiate Faculty of Biotechnology, University of Gdańsk and Medical University of Gdańsk, 80-307 Gdańsk, Poland; 7 Center of Biostatistics and Bioinformatics, Medical University of Gdańsk, 80-211 Gdańsk, Poland; 8 Department of Pathomorphology, Faculty of Medicine, Medical University of Gdańsk, 80-210 Gdańsk, Poland; aleksandra.ciarka@gumed.edu.pl; 9 2nd Division of Radiology, Faculty of Health Sciences with the Institute of Maritime and Tropical Medicine, Medical University of Gdańsk, 80-210 Gdańsk, Poland; jaziel@gumed.edu.pl

**Keywords:** gastric cancer, *HER2* amplification, HER2 overexpression, microRNA profiling, chromosome 17 polysomy, prognostic biomarkers

## Abstract

Human epidermal growth factor receptor 2 (HER2) remains the most recognized and clinically established molecular biomarker in gastric cancer; however, the regulatory mechanisms underlying its dysregulation are not fully understood. This study aimed to identify microRNAs associated with *HER2* gene amplification, chromosome 17 centromere copy number increase (CNI), or alternative mechanisms driving HER2 protein overexpression. We analyzed 115 gastric cancer patients treated surgically at a single institution, with available material for immunohistochemistry (IHC), fluorescence in situ hybridization (FISH), and microRNA profiling. Among 11 candidate microRNAs, four demonstrated significant associations with HER2-related alterations. hsa-miR-128-3p expression was positively associated with *HER2* gene amplification, while hsa-miR-145-5p expression showed an inverse relationship with centromere enumeration probe 17 (*CEP17*) signal count and correlated with membranous HER2 protein expression. hsa-miR-27b-5p expression was linked to *CEP17* CNI, whereas hsa-miR-552-3p expression was associated with both increased *HER2* amplification and *CEP17* signal count. Importantly, hsa-miR-27b-5p upregulation independently predicted worse overall survival, whereas hsa-miR-128-3p upregulation independently predicted improved survival outcomes. These findings identify distinct microRNA signatures associated with HER2 pathway alterations and prognosis in gastric cancer, highlighting their potential as biomarkers and contributors to HER2-driven tumor biology.

## 1. Introduction

Gastric cancer remains one of the leading causes of cancer-related mortality worldwide [1]. Because of prevalent late-stage diagnosis and highly probable relapse after up-front surgery, combined modality therapies are standard for stage ≥ IB disease [2].

Among the many molecular markers that have been identified to improve treatment outcomes, HER2 has been shown to have the greatest clinical significance [3]. The *HER2* gene, also known as *ERBB2* or *HER2*/*neu* is a proto-oncogene located in the long arm of chromosome 17 that encodes the transmembrane tyrosine receptor HER2, which plays a critical role in cellular signal transduction in numerous cancers [4]. In case of too many copies of *HER2* gene resulting in overexpression of HER2 protein, the excessive activation of signaling pathways can lead to uncontrolled cell division, proliferation, differentiation, inhibition of apoptosis, angiogenesis, lymphangiogenesis and metastasis formation [3,4,5,6,7].

HER2 positivity of gastric cancer occurs in approximately 10–20% of cases and constitutes an indication for trastuzumab-based therapy [2,8]. Trastuzumab (Herceptin, Genentech) is a humanized monoclonal antibody that directly binds to the extracellular domain of the transmembrane HER2 receptor and inhibits the proliferation and survival of HER2-overexpressing cancer cells [9]. However, the response rate to trastuzumab therapy ranges from 32% to 68% [3,7,8]. Several other immune checkpoint inhibitors have also been investigated in HER2-positive gastric cancer; however due to limited improvement in clinical outcome, there is still no consensus regarding which treatment strategy may be superior for specific patients and disease conditions [3,7]. The efficacy of biological treatment is further limited by factors such an intratumoral heterogeneity of HER2 expression in gastric adenocarcinoma [2]. Some other mechanisms of resistance to trastuzumab-based treatment have been hypothesized; however, in clinical practice they remain largely unpredictable [7,10].

The discovery of microRNAs and their role in gene regulation has recently been recognized by the Nobel Prize Committee with the Prize awarded jointly to Victor Ambros and Gary Ruvkun [11]. Under physiological conditions, microRNAs are responsible for the homeostatic regulation of gene expression and for cellular adaptation to transient changes in the cell microenvironment [12,13]. They also play a significant role in tumorigenesis by altering the expression of oncogenes and tumor suppressors through messenger RNA degradation and post-transcriptional inhibition, thereby influencing key cellular pathways including proliferation, differentiation, apoptosis and drug sensitivity [10]. Dysregulation of various microRNAs has been reported in nearly all types of cancer, and each tumor type may exhibit a characteristic and unique microRNA expression signature [13].

The possibility of modulating microRNA activity to reverse their oncogenic effects has prompted researchers worldwide to investigate these molecules as potential diagnostic and therapeutic biomarkers in numerous types of cancer, including gastric cancer [13,14]. However, so far the role of microRNAs in the regulation of the HER2 signaling network remains poorly understood [15].

Despite increasing incidence on the role of microRNAs in gastric cancer biology, their potential involvement in the regulation of HER2-related molecular alterations, including *HER2* gene amplification and chromosome 17 copy number changes, remains insufficiently explored.

Importantly, HER2 status assessment in gastric cancer is influenced not only by gene amplification but also by chromosomal alterations such as chromosome 17 centromere copy number increase (CNI), which may affect the interpretation of FISH results and potentially lead to misclassification of HER2 status [5,16]. Therefore, investigating upstream regulatory mechanisms, including microRNA expression, may provide additional insight into HER2 pathway dysregulation.

The present study should be interpreted as a secondary analysis of a previously characterized gastric cancer cohort. Earlier investigations from this cohort addressed the impact of chromosome 17 centromere enumeration probe (*CEP17*) CNI on survival and the association of selected microRNAs with lymphatic spread [17]. In contrast, the current study focuses on a distinct research question, namely the relationship between microRNA expression and HER2-related molecular alterations, including HER2 protein expression, *HER2* gene amplification and *CEP17* copy number increase.

The aim of this study was to identify specific microRNAs associated with HER2-related molecular features and to evaluate their potential prognostic significance in gastric cancer.

The identification of microRNAs that play a role in *HER2* gene amplification or in other mechanisms leading to HER2 protein overexpression could provide new insights into the development of novel biologically targeted therapies for HER2-dependent gastric cancers, beyond trastuzumab-based strategies. It may also contribute to the development of approaches aimed at overcoming resistance to trastuzumab-based therapy.

## 2. Results

### 2.1. Characteristics of the Studied Group

The mean (median) age of the patients was 62.7 (63) years. The cohort comprised 82 (71.3%) males and 33 (28.7%) females. The cardia was involved in 41 (36.7%) cases, while the remaining lesions were found in the distal stomach. Detailed clinicopathological characteristics and survival data are presented in Table 1.

### 2.2. The Correlations Between microRNAs Expression and HER2

Regarding IHC results, upregulation of hsa-miR-145-5p was the only microRNA associated with IHC 3+ status, both in the unadjusted model (OR 1.86, 95% CI 1.05–3.62; *p* = 0.0329) and in the fully adjusted model (OR 2.16, 95% CI 1.06–5.18; *p* = 0.0344). The remaining microRNAs were not associated with IHC status, or their potential associations did not reach statistical significance (Table 2).

Correlations between microRNA expression and the parameters measured in fluorescence in situ hybridization (FISH) test result are presented in Table 3. Among the analyzed microRNAs, hsa-miR-128-3p showed the strongest associations with quantitative FISH measures, demonstrating positive correlations with all assessed FISH parameters. Lower expression of hsa-miR-145-5p was associated with higher *CEP17* signals, but not with *HER2* signals or the *HER2*/*CEP17* ratio. In contrast, higher hsa-miR-552-3p expression was strongly positively correlated with *CEP17* and *HER2* signals, but not with the ratio. No significant correlations were observed for hsa-miR-27b-5p or the other analyzed microRNAs. In analyses based on log2-transformed data, the results were largely consistent. The most robust associations involved higher expression of hsa-miR-552-3p and hsa-miR-128-3p with increased absolute signal counts (*CEP17* and/or *HER2*), whereas only hsa-miR-128-3p was associated with the *HER2*/*CEP17* ratio.

hsa-miR-27b-5p was associated with *CEP17* CNI in both the unadjusted model (OR 1.86, 95% CI 1.06–3.53; *p* = 0.0293) and the fully adjusted model (OR 1.95, 95% CI 1.10–3.78; *p* = 0.0215). No analyzed microRNA showed a significant association with the presence of FISH amplification or composite HER2 status in the adjusted analysis (Table 2).

### 2.3. Correlations Between miRNAs Expression and Clinicopathological Parameters

As only four of the 11 analyzed microRNAs showed significant associations with IHC or FISH results, further analyses of correlations with clinicopathological parameters and survival were restricted to hsa-miR-27b, hsa-miR-128-3p, hsa-miR-145-5p, and hsa-miR-552-3p. Among the 115 cases, successful assessment was achieved for all specimens only for hsa-miR-145-5p. Expression data were obtained for 74 cases for hsa-miR-27b, 114 for hsa-miR-128-3p, and 84 for hsa-miR-552-3p.

hsa-miR-145-5p showed the broadest range of clinicopathological associations. Its expression was lower in pT3–4 than in pT1–2 tumors, lower in the tumors located proximally in the stomach, and lower in non-mucinous compared with mucinous tumors. It also varied across Lauren subtypes, with the lowest levels observed in intestinal-type and the highest in diffuse-type cancers. hsa-miR-128-3p expression was higher in stage III–IV disease, lower in mucinous than in non-mucinous tumors, and also differed across Lauren subtypes, with the lowest levels in diffuse-type tumors. hsa-miR-552-3p expression likewise varied by Lauren subtype, whereas hsa-miR-27b-5p showed no significant clinicopathological associations (Table 4).

### 2.4. The Correlations Between miRNAs Expression and Survival

**Figure 1:**
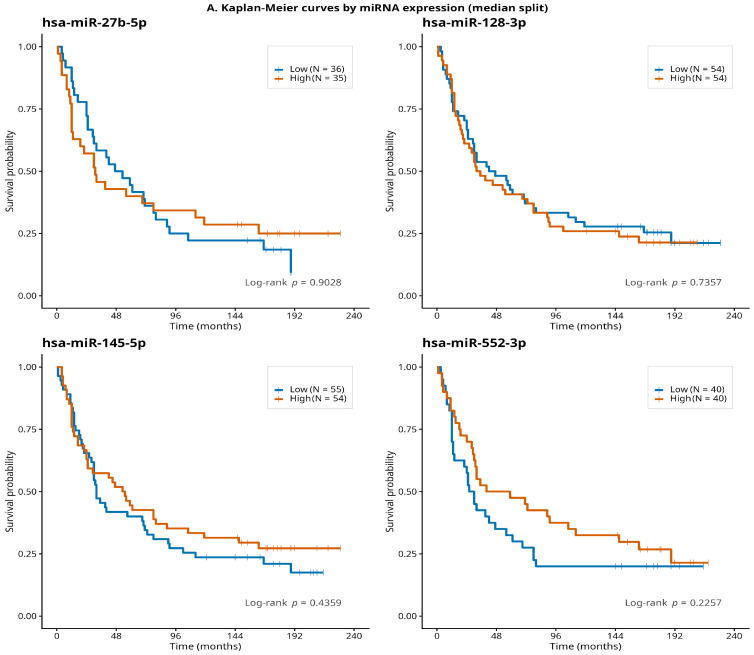

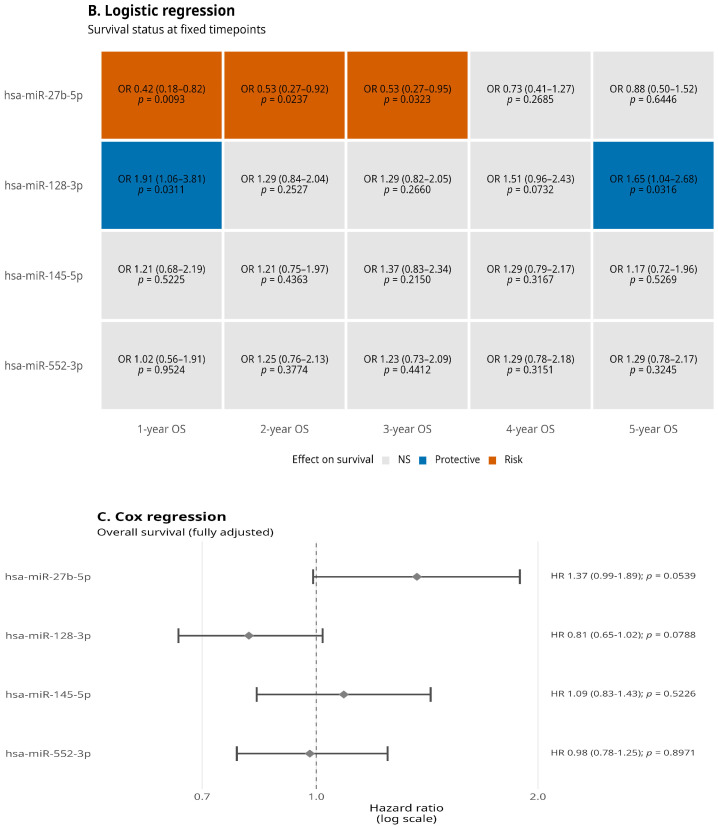
Association between hsa-miR-27b, hsa-miR-128-3p, hsa-miR-145-5p and hsa-miR-552-3p and survival. (**A**) Kaplan-Meier curves by miRNA expression (median split); (**B**) Logistic regression (**C**) Cox regression.

Kaplan–Meier curves based on median dichotomization did not show significant separation for any of the four microRNAs analyzed (Figure 1). However, regression analyses suggested opposite directions of association for hsa-miR-27b-5p and hsa-miR-128-3p. After adjustment, higher expression of hsa-miR-27b-5p was associated with lower odds of survival at 1, 2, and 3 years, whereas higher expression of hsa-miR-128-3p was associated with higher odds of survival at 1 and 5 years.

Higher hsa-miR-145-5p expression was associated with improved 3-, 4-, and 5-year survival only in unadjusted analyses, while hsa-miR-552-3p showed no association with survival at any time point. Cox regression analyses demonstrated a consistent pattern: higher hsa-miR-27b-5p expression was associated with worse overall survival (HR 1.40, 95% CI 1.03–1.90), whereas higher hsa-miR-128-3p expression was associated with better overall survival (HR 0.79, 95% CI 0.63–0.99) (Table 5 and Table 6; Figure 1).

**Figure 2:**
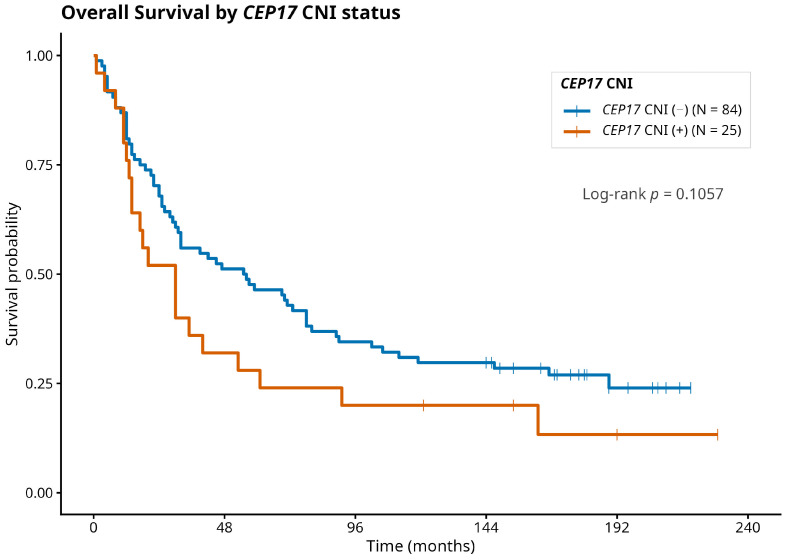
Comparison of overall survival rates of patients with the presence or absence of *CEP17* CNI.

Analysis of the association between the presence or absence of CEP17 CNI and survival revealed a trend toward differences in survival rates; however, these differences did not reach statistical significance at any of the analyzed time points (*p* > 0.05). A trend toward lower survival rates in the *CEP17* CNI–positive group compared to the *CEP17* CNI–negative group was most pronounced at the 3-year follow-up (*p* = 0.08) (Table 7 and Figure 2).

## 3. Discussion

**Figure 3:**
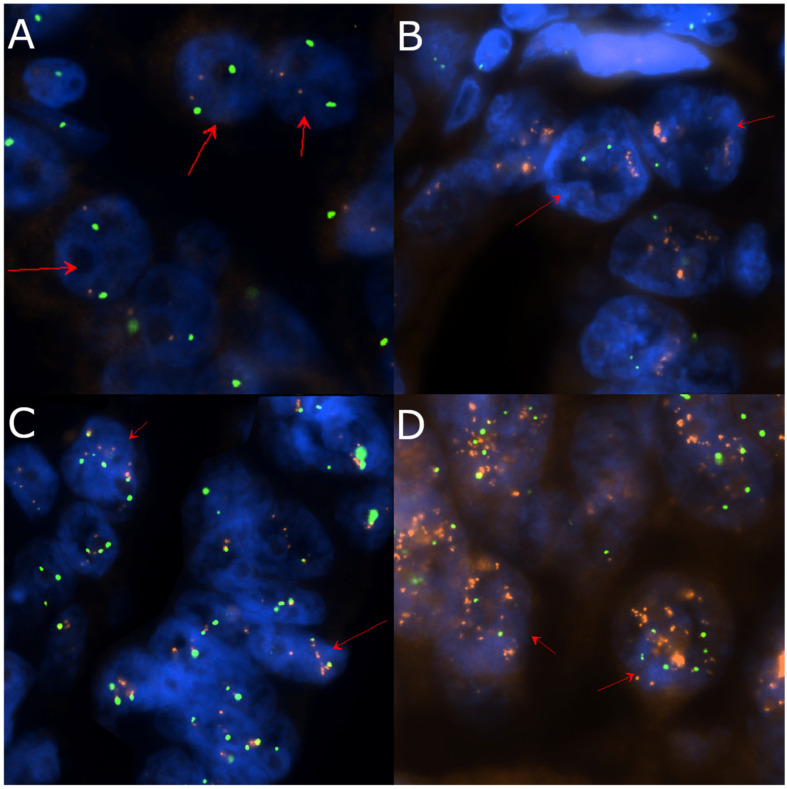
Dual-probe FISH assays demonstrating *HER2* gene copies (red) and *CEP17* (green). (**A**) FISH-negative result (no amplification). Arrows indicate cells with two *HER2* signals and two *CEP17* signals. (**B**) FISH-positive result (amplification). Arrow indicates a cell with multiple *HER2* signals and two *CEP17* signals. (**C**) *CEP17* CNI, FISH negative. Arrows indicate cells with 3–4 *HER2* signals and 3–4 *CEP17* signals. (**D**) *CEP17* CNI and *HER2* gene amplification. Arrows indicate cells with multiple *HER2* and *CEP17* signals.

HER2 status in gastric cancer is primarily determined by immunohistochemical assessment of membranous HER2 protein expression [16]. In cases with equivocal IHC scores (2+), HER2 gene amplification is subsequently evaluated using FISH typically employing a dual-probe assay in which one probe detects *HER2* gene copy number (red signals) and the second targets the centromeric region of chromosome 17 (*CEP17*; green signals), where the *HER2* gene is located (Figure 3) [5,16]. A result is considered positive when the *HER2*/*CEP17* ratio exceeds 2 [5,16]. Consequently, an increased number of chromosome 17 copies may potentially lead to false-negative or false-equivocal FISH result [5]. Therefore, understanding molecular mechanisms that influence both *HER2* gene copy number and *CEP17* alterations is of particular importance, as these factors may contribute to variability in HER2 status assessment and therapeutic response.

It is worth noticing that true chromosome 17 polysomy is extremely rare [18,19]. In most cases, the observed increase reflects amplification of the pericentromeric region of chromosome 17, including the *HER2* locus [18,19]. Therefore, this phenomenon should be referred to not as chromosome 17 polysomy but rather as an increase in centromere 17 copy number [18,19].

Because the ratio of *HER2* gene copies to *CEP17* signals is rarely below 1, it can be assumed that, in practice, each additional *CEP17* signal corresponds to an additional *HER2* gene copy. In our 2021 study, we demonstrated that *CEP17* CNI was strongly associated with HER2 protein overexpression and had a greater impact on patient outcomes than HER2 status itself [5]. In that study, performed on 244 patients, *CEP17* CNI was observed in 17.2% of cases, compared with 23.5% in the present cohort. This difference is attributable to the modified exclusion criteria applied here, specifically the additional exclusion of patients who received preoperative chemotherapy, which altered the clinicopathological composition of the study group.

Therefore, it is important to investigate genetic factors influencing not only HER2 status per se, but also the individual parameters of the FISH assay. In our analysis, we identified statistically significant associations between the expression of four microRNAs and these parameters. Notably, each microRNA exhibited a distinct pattern of association with the FISH-derived parameters.

A positive correlation between miR-128-3p expression and *HER2* signals, *CEP17* signals, and the *HER2*/*CEP17* ratio suggests a role for miR-128-3p in *HER2* gene amplification without a corresponding effect on *CEP17* copy number. Upregulation of miR-145-5p was associated with a decrease in *CEP17* copy number without affecting *HER2* gene amplification. In contrast, upregulation of miR-27b-5p was associated with the presence of *CEP17* CNI. Notably, miR-552-3p expression was positively associated with both *HER2* and *CEP17* signals, without affecting the *HER2*/*CEP17* ratio.

Regarding IHC results, upregulation of miR-145-5p was the only microRNA significantly associated with IHC 3+ status. The remaining miRNAs were not associated with IHC status, or their potential associations did not reach statistical significance.

The role of miR-145-5p in gastric cancer was extensively discussed in our previous report on the association of microRNAs with lymphatic spread [17]. In that study, miR-145-5p was an independent positive prognostic factor for overall survival (HR 0.78; *p* = 0.025). In the present analysis, this association was not confirmed in the adjusted Cox model (HR 1.04; *p* = 0.76), likely reflecting differences in cohort composition following the application of additional exclusion criteria and the use of a different covariate structure in the regression models. Consistent with our previous findings, miR-145-5p expression was associated with several clinicopathological parameters; however, in the present study, we focused specifically on its relationship with HER2-related alterations rather than re-evaluating its clinical associations. Notably, we observed an inverse relationship between miR-145-5p expression and *CEP17* copy number, suggesting a potential role of this microRNA in regulating chromosomal alterations rather than directly influencing *HER2* gene amplification. It may represent an additional mechanism underlying its well-recognized tumor-suppressive effect.

Only a limited number of studies have investigated the potential relationship between miR-145 expression and the HER2 receptor in breast cancer [20,21], and to the best of our knowledge, no such studies have been reported in gastric cancer.

The experimental findings regarding the role of miR-27b in tumorigenesis reported in the literature remains inconsistent [22,23,24,25]. An in vitro study demonstrated that miR-27b promotes proliferation, invasion and metastasis of gastric cancer cells by downregulating ADAMTS5, a protease involved in proteoglycan degradation and acting as a tumor suppressor in gastric cancer [22]. In contrast, other in vitro and in vivo studies have reported a tumor-suppressive role of miR-27b, inhibiting both lymphatic and liver metastasis of gastric cancer through downregulation of nuclear receptor subfamily 2 (NRF2F2) [23]. Overexpression of miR-27b also suppressed proliferation, migration, and invasion in the MGC-803 gastric cancer cell line and reduced colony formation in human gastric epithelial cells (GES-1) [23]. The tumor-suppressive role of miR-27b in gastric cancer progression was further supported by a study showing that miR-27b-3p directly targets the oncogene ROR1, leading to its downregulation [25]. Moreover, SGC7901 cells transfected with miR-27b exhibited significantly increased apoptosis and reduced proliferation [26]. Interestingly, in colorectal cancer, exosomal miR-27b-3p has been reported to enhance vascular permeability by down-regulating vascular endothelial cadherin (VE-Cad) and p120 catenin, thereby promoting metastatic dissemination [24].

In our study, miR-27b-5p overexpression was significantly associated with the presence of *CEP17* CNI, which represents a novel finding not previously reported in gastric cancer. This observation may suggest a potential link between microRNA expression and chromosomal instability mechanisms, which could indirectly influence HER2 status assessment. Upregulation of miR-27b-5p was not associated with any clinicopathological parameters, but it appeared to be an independent negative prognostic factor for survival. Interestingly, this microRNA was positively correlated with the presence of *CEP17* CNI, which we previously demonstrated to be a potential (*p* = 0.05) negative prognostic factor for 2-year survival in gastric cancer patients [5]. In current cohort, a similar trend was observed, with *CEP17* CNI associated with poorer survival, although the association did not reach statistical significance (Figure 2, Table 7).

Similar to miR-145, no studies have yet investigated the relationship between miR-27b and HER2 in gastric cancer, with only a few studies addressing this relationship in breast cancer [27,28].

The role of miR-128 is well established in both physiological and pathological processes [29]. It plays a critical role in nervous system development and in maintaining essential functions such as cognition and memory [29,30]. miR-128 is also recognized as a tumor suppressor in multiple cancers, including brain tumors, prostate, pancreatic, colon, gastric, ovarian, and breast cancers [29,30]. While previous studies have focused primarily on its role in proliferation and survival, our findings suggest that miR-128-3p may also be involved in *HER2* gene amplification-related processes, as indicated by its positive correlation with *HER2* gene copy number and *HER2*/*CEP17* ratio.

Owing to its diverse functions and mechanisms of tumor suppression, miR-128 has been proposed as a potential target for cancer prevention and therapy [30]. For example, in breast cancer, overexpression of miR-128-3p has been shown to significantly inhibit cancer cell proliferation and is associated with improved prognosis [31].

In gastric cancer, one in vitro study demonstrated that miR-128-3p may be transported via exosomes to the tumor microenvironment, where it can facilitate angiogenesis [32]. Interestingly, another study reported that miR-128, miR-27b, and miR-101 inhibited angiogenesis through direct downregulation of VEGF-C expression [33]. The authors did not observe any association between miR-128 or miR-27b expression and patient survival, although the cohort included only 48 patients [33]. In the same study, both miR-128 and miR-27b were found to significantly reduce the migration and invasion capacity of MKN-45 gastric cancer cells [33].

A clinical study involving 103 patients confirmed our findings regarding the association between high miR-128-3p expression, lower TNM stage, and improved survival [34]. The experimental part of that study demonstrated that miR-128-3p inhibits the expression of the oncogenic protein Tuftelin1, which promotes cancer cell viability, invasion, epithelial-mesenchymal transition, and suppresses apoptosis [34]. In our cohort, we observed an opposite association between miRNA-128-3p expression and TNM stage and confirmed that up-regulation of miR-128-3p is an independent prognostic factor for improved survival. Furthermore, higher miR-128-3p expression was associated with intestinal-type tumors according to Lauren classification and non-mucinous histology. Other studies have reported that miR-128 suppresses proliferation and promotes apoptosis through alternative mechanisms, such as downregulation of the oncogenic protein GAREM (Grb2-associated regulator of ERK/MAPK1) [35]. Improved survival of gastric cancer patients with high miR-128 expression was also demonstrated in a study identifying a prognostic signature of four microRNAs (miR-128, miR-27b, miR-100, and miR-214) predictive of lymph node metastasis [36]. In our analysis, upregulation of miR-128-3p was additionally associated with increased *HER2* gene amplification. It is worth noticing that unlike in breast cancer, the prognostic significance of HER2 status in gastric cancer remains controversial [16].

miR-552 is overexpressed and functions as a tumor promoter in various human cancers [37]. In contrast to prior studies linking miR-552 to aggressive tumor behavior, we observed its association primarily with *HER2* and *CEP17* signal counts, without a clear impact on survival, suggesting that its role may be more related to genomic alterations than to direct clinical outcomes. It enhances tumorigenesis by targeting multiple genes with diverse molecular functions and may contribute to resistance to anticancer therapies [37]. In a study of 183 colorectal cancer patients, upregulation of miR-552 was associated with higher histological grade, lymph node metastasis, advanced TNM stage, and poorer overall survival [38]. In gastric cancer, a study of 122 specimens demonstrated that miR-552 overexpression correlated with more advanced disease stage, lymph node metastasis, intestinal metaplasia, genomically stable tumor type, and worse survival [39]. In the experimental part of the study miR-552 enhanced cells proliferation migration and invasion of gastric cancer cells [39]. In our cohort, however, upregulation of miR-552-3p was associated only with intestinal-type tumors and showed no significant correlation with other clinicopathological parameters or survival.

*HER2* amplification results in persistent activation of the HER2 receptor, which promotes cancer growth through multiple intracellular signaling pathways, including the mitogen-activated protein kinase (MAPK), phosphatidylinositol-3 kinase (PI3K)/AKT, and mTOR pathways [40]. Each microRNA has the potential to target multiple mRNAs and thereby regulate numerous genes, often involved in the same tumor-promoting mechanisms [13,40]. In vitro studies have demonstrated that it is possible to reverse the effects of oncogenic microRNAs or to enhance microRNA-mediated tumor suppression [31,32,33,39]. Therefore, the identification of microRNAs regulating HER2-dependent tumorigenic pathways represents an attractive strategy for the development of novel targeted therapies.

Such relationships have been described in breast cancer [40]. The authors identified 38 microRNAs as inhibitors of HER2 signaling and also found 7 microRNAs (including miR-552) that directly target HER2 expression [40]. In the same study, miR-342 was shown to inhibit the growth of HER2-positive cells and was associated with improved survival in the overall breast cancer population [40]. Another study demonstrated that retroviral upregulation of miR-125a and its homolog miR-125b suppresses HER2 signaling, resulting in reduced migration and invasion capacities in HER2-dependent breast cancer cell lines [41].

miR-125a-5p has also been shown to directly target HER2 in gastric cancer [42]. Suppression of gastric cancer cell lines proliferation was more pronounced when combined with the addition of trastuzumab solution [42]. In the clinical part of the study, which included 52 gastric carcinoma specimens, downregulation of miR-125a-5p was associated with advanced tumor stage, high HER2 protein expression, and poor prognosis [42]. In the present study, both homologs of miR-125 were among the most stably expressed microRNAs based on NGS analysis and were therefore used as reference microRNAs for qPCR normalization. However, no significant associations were observed between miR-125 expression and any clinicopathological parameters in the overall cohort.

Further evidence for microRNA-mediated regulation of HER2 has been reported in gastric cancer [43,44,45]. A study involving 30 gastric cancer cases demonstrated an association between HER2 status and miR-375 expression and showed that miR-375 directly targets *HER2* gene expression [43]. However, in the present study, this relationship was not confirmed in our cohort of 115 patients. In addition, other in vitro studies have suggested that resistance to trastuzumab in gastric cancer cell lines may be overcome by suppression of specific microRNAs, such as miR-223 or miR-21 [44,45].

Although direct regulation of chromosome 17q12 genes by the investigated microRNAs has not yet been conclusively demonstrated and requires further experimental validation, we explored the miRDB database to determine whether miR-27b-5p, miR-128-3p, miR-145-5p, and miR-552-3p possess predicted binding sites within genes located in the 17q12 amplicon, including ERBB2, GRB7, STARD3, PGAP3, and MIEN1.

Bioinformatic analysis using the miRDB database identified 1254 predicted target genes for hsa-miR-128-3p, 836 for hsa-miR-552-3p, 266 for hsa-miR-27b-5p, and 909 for hsa-miR-145-5p. Functional in silico analysis of the predicted targets revealed enrichment of genes involved in pathways relevant to HER2-positive gastric cancer, including receptor tyrosine kinase signalling, *PI3K*/*AKT*, *MAPK*/*ERK*, cell-cycle regulation, epithelial–mesenchymal transition (EMT), angiogenesis, and transcriptional control. Among the predicted targets of miR-128-3p were genes associated with transcriptional regulation and tumor suppression, including *AFF4* and *SZRD1*, suggesting indirect modulation of HER2 downstream signalling. For miR-552-3p, several oncogenic drivers were identified, including *CDK6*, *WNT9B* and *FOXC1* relevant to cancer biology. Although *ERBB2* (*HER2*) and *ERBB3* (*HER3*) were not identified as direct predicted targets, several downstream effectors and pathway components associated with receptor tyrosine kinase signaling were represented among the predicted targets, suggesting that miR-552-3p may indirectly influence HER2-positive tumor biology through modulation of interconnected oncogenic networks. Similarly, miR-145-5p was predicted to target multiple cancer-related genes, including *ERBB4*, *IGF1R*, *AKT3*, *CDK6*, *NRAS*, *CRKL*, *YES1*, and *PAK1*, all of which participate in signalling pathways converging on *PI3K*/*AKT* and *MAPK* cascades.

In contrast, the predicted target profiles of miR-27b-5p included genes implicated in cellular proliferation, migration, and signal transduction, further supporting its potential involvement in gastric cancer progression.

Taken together, the predicted target profiles of miR-128-3p, miR-552-3p, miR-27b-5p, and miR-145-5p suggest that these microRNAs may affect multiple biological processes relevant to gastric cancer progression, including cell-cycle regulation, transcriptional control, EMT, and *PI3K*/*AKT*- and *MAPK*-related signaling pathways. Importantly, none of the analyzed microRNAs was predicted to directly target *ERBB2*, suggesting that their association with the HER2-positive phenotype may result from indirect regulation of downstream signalling components and interacting molecular networks. This interpretation is consistent with the survival analyses and Cox regression models presented for miR-27b-5p and miR-128-3p, where altered expression of these microRNAs was associated with patient outcome.

Nevertheless, it should be emphasized that the identified microRNA–mRNA interactions are based solely on computational target predictions generated using miRDB and therefore require further functional and experimental validation.

Accordingly, further studies, including gene expression analyses, luciferase reporter assays, and in vitro or in vivo validation experiments, are required to confirm the biological significance of these predicted interactions and to clarify their mechanistic contribution to HER2/HER3-associated gastric cancer progression. In addition, validation of the identified miRNA signatures in large, prospective, multicenter cohorts will be necessary to establish their clinical relevance and generalizability. Given the growing interest in circulating and exosomal miRNAs as minimally invasive biomarkers [46,47,48], future studies should investigate whether the miRNAs identified in the study could serve as liquid biopsy biomarkers for the detection and characterization of HER2-positive gastric cancer.

The present study has several limitations that should be acknowledged. First, it was an exploratory retrospective biomarker analysis performed in a relatively small cohort, and multiple related hypotheses were examined without formal multiplicity correction. Therefore, statistically significant findings should be interpreted as hypothesis-generating rather than confirmatory. Second, Kaplan–Meier plots based on median split were used primarily for visualization of survival patterns; the main inferential estimates are provided by regression models using continuous miRNA expression scaled per 1 standard deviation of log2-transformed values. Third, the observed associations require validation in an independent cohort before any firm biological or clinical conclusions can be drawn. Due to the purely clinical nature of our study, we were unable to elucidate the molecular mechanisms underlying the relationships between microRNA expression and *HER2* gene amplification or *CEP17* CNI. The study as a clinical and exploratory biomarker analysis does not include experimental in vitro or mechanistic validation. Therefore, the observed associations between microRNA expression and HER2-related molecular alterations should not be interpreted as evidence of direct causal regulation.

The observed trend toward poorer survival in cases with *CEP17* CNI may be associated with either HER2-related mechanisms or the influence of other oncogenesis-related genes located in the pericentromeric region of chromosome 17 (e.g., *TOP2A*, *DARPP32*, *BRCA1*, *TP53*), as previously suggested in our earlier report [5]. The present study is also based on a previously analyzed cohort, which may introduce a degree of overlap with earlier publications. However, the current analysis addresses a distinct biological question and applies a different analytical framework focused on HER2-related molecular parameters.

To the best of our knowledge, this is the first clinical study to comprehensively evaluate the associations between selected microRNAs and HER2-related molecular alterations, including *HER2* gene amplification and *CEP17* CNI in gastric cancer. In our findings distinct microRNAs were differentially associated with specific components of HER2 assessment, suggesting that microRNA-mediated regulation may contribute not only to HER2-driven signaling but also to broader genomic alterations involving chromosome 17. We demonstrated a positive association between miR-128-3p expression and *HER2* gene amplification, a negative association between miR-145-5p expression and *CEP17* signal count, an association between miR-27b-5p and the presence of *CEP17* CNI, and a relationship between miR-552-3p expression and both *HER2* amplification and *CEP17* signals. An interesting finding is that high expression of miR145-5p was associated with a low number of CEP17 signals, while at the same time correlating with high expression of the membrane receptor HER2 on the surface of cancer cells. Importantly, we identified upregulation of miR-27b-5p as an independent negative prognostic factor, whereas miR-128-3p upregulation was found to be an independent positive prognostic factor for overall survival in gastric cancer patients.

These findings support the potential biological and clinical relevance of HER2-associated microRNA profiles in gastric cancer and provide a rationale for further translational and mechanistic studies.

## 4. Materials and Methods

The study included 144 consecutive patients who underwent elective major gastric resection for gastric adenocarcinoma between 1 August 2006 and 31 December 2013 at the Department of Surgical Oncology, Medical University of Gdańsk. Exclusion criteria were the presence of another malignant gastric tumor (other than adenocarcinoma) and preoperative chemotherapy.

Clinicopathological data of the patients were retrospectively retrieved, and the pathological specimens were thoroughly reexamined at the Department of Pathomorphology, Medical University of Gdańsk. Patient consent was waived by the Ethics Committee of the Medical University of Gdańsk. In addition to routine histopathological reevaluation, all samples were subjected to assessment of HER2 protein expression by immunochemistry (IHC).

Tumor tissue was additionally collected for genetic analyses. Both *HER2* gene amplification assessed by fluorescence in situ hybridization (FISH) and microRNA assays were performed at the Department of Molecular Oncology and Genetics, Innovative Medical Forum, Łukaszczyk Oncology Centre in Bydgoszcz.

The main parameters of interest included HER2 protein expression assessed by IHC, the presence of *HER2* gene amplification evaluated by FISH, HER2 status determined according to the guidelines of the College of American Pathologists, the American Society for Clinical Pathology, and the American Society of Clinical Oncology (CAP/ASCP/ASCO) [16], the presence of *CEP17* copy number increase (CNI), the number of *HER2* gene signals per nucleus, the number of *CEP17* signals per nucleus, and the *HER2*/*CEP17* ratio determined by FISH.

HER2 status was considered positive in cases with IHC results of 3+, or 2+ with the presence of *HER2* gene amplification (FISH-positive) [16]. *CEP17* CNI, previously referred to as chromosome 17 polysomy, was defined as the presence of ≥3 CEP17 signals [5,16].

Routine histopathological re-examination focused on the depth of tumor invasion in the gastric wall (pT), the number of positive lymph nodes (pN), and the pTNM stage according to the 8th edition of the AJCC Cancer Staging Manual [49]. Additional parameters included Lauren histological tumor type, the presence of a mucinous component in the tumor tissue, and tumor location within the stomach. Survival data were obtained from the Polish Ministry of Digitalization on 2 December 2025.

The study was approved by the Independent Ethics Committee of the Medical University of Gdańsk (NKBBN/90/2017).

Among 144 primary tumor specimens, 28 were excluded from the current analysis due to previous chemotherapy (12 cases), insufficient tumor material (15 cases), or concomitant gastric lymphoma (1 case). Due to failure of microRNA assessment in one specimen, the final study cohort consisted of 115 cases.

For statistical purposes, patients with pT1–2 stages were grouped together, as were those with pT3–4 stages. Similarly, TNM stages I–II and III–IV were combined into two groups. To assess the relationship between microRNA expression and IHC results, IHC scores of 0, 1+, and 2+ were classified as negative, while a score of 3+ was considered positive.

The methodologies of the IHC and FISH assays were described in detail in our previous paper concerning the impact of chromosome 17 centromere CNI on gastric cancer patient survival [5]. The expression of microRNAs in gastric cancer was of primary interest to us due to their potential influence on lymphatic dissemination. We considered it worthwhile to further explore this topic. Therefore, in the present study, we analyzed the association of these previously examined microRNAs with HER2 receptor expression, *HER2* gene amplification, and chromosome 17 copy number. The methodology of microRNA assays has been described in detail in our previous report [17].

### 4.1. Immunohistochemistry (IHC)

IHC staining was performed on 4-μm sections of paraffin-embedded tumor tissue, with representative areas selected to avoid necrosis. The staining procedure and evaluation criteria were identical to those described in our previous study on CEP17 copy number increase [5]; therefore, only a concise description is provided here.

The procedure, including deparaffinization, antigen retrieval, and HER2 staining, was carried out using an automated system (Roche Benchmark GX, ROCHE, Basel, Switzerland) following standard protocols and the manufacturer’s instructions, as previously described [5]. Sections were counterstained with hematoxylin and evaluated under the Olympus BX43 light microscope (magnification, ×40; Olympus Corporation, Tokyo, Japan) according to the criteria recommended by Hofmann et al. [50].

### 4.2. Fluorescence In Situ Hybridization (FISH)

Molecular cytogenetic (FISH) analysis was performed on 4–6 μm sections of paraffin-embedded tumor tissue at the Molecular Oncology and Genetics Department, Innovative Medical Forum, Łukaszczyk Oncology Centre in Bydgoszcz. Representative tumor areas were selected to avoid necrosis. *HER2* gene amplification was assessed using the commercially validated Vysis PathVysion *HER2* FISH kit (Abbott Pharmaceutical, Abbott Park, IL, USA) following the manufacturer’s protocol and standard CAP/ASCP/ASCO 2016 recommendations [16]. At least 60 interphase nuclei per sample were evaluated under a fluorescence microscope, and the *HER2*/*CEP17* ratio and average copy numbers were reported by a qualified cytogeneticist. Results were interpreted as positive (*HER2*/*CEP17* ratio ≥ 2) or negative (ratio < 2), with additional criteria applied for cases with *CEP17* CNI, defined as ≥3 *CEP17* copies per cell). In cases with *CEP17* CNI and a ratio < 2, the presence of >6 *HER2* signals was interpreted as a positive result, <4 *HER2* signals was interpreted as a negative result, and a signal number between 4 and 6 was interpreted as an equivocal result, as described previously [5,17]. Images were captured using Lucia Cytogenetics 2 Laboratory Imaging software v.2.1 (Lucia Cytogenetics, Praha, Czech Republic), examples of which are presented in Figure 3.

### 4.3. microRNA Analysis

Isolation of microRNA derived from 32 gastric adenocarcinoma patients (three groups: pT1-T2N1, pT3-T4N0 and pT3-T4N3) was performed using the miRCURY RNA isolation kit dedicated for FFPE samples (Exiqon, Qiagen, Copenhagen, Denmark), and quality and quantity control were performed (Bioanalizator, Agilent, Santa Clara, CA, USA). Based on quality metrics, 10 of 32 samples representing all experimental groups were selected for next-generation sequencing (NGS) using the Illumina NextSeq 500 platform, (Ilumina Inc., San Diego, CA, USA) as previously described [18]. NGS data were processed for adapter trimming, quality control, and read assignment. The most stably expressed microRNAs were identified with NormFinder and used as references for qPCR normalization. We chose *hsa-miR-30c-5p*, *hsa-miR-125b-5p* and *has-miR125a-5p* based on the NGS experimental approach and *U6*, *UniSP3* and *UniSp6* as control microRNAs for qPCR normalization. To construct the custom qPCR panel for analysis in the whole cohort, three microRNAs (hsa-miR-196a-5p, hsa-miR-552-3p, and hsa-miR-708-3p) demonstrating the greatest differences in expression between the analyzed groups were selected based on the preliminary screening phase. Eight additional microRNAs (hsa-miR-375, hsa-miR-145-5p, hsa-miR-21-5p, hsa-miR-187-3p, hsa-miR-142-3p, hsa-miR-27b-5p, hsa-miR-382-5p, and hsa-miR-128-3p) were selected based on literature data indicating their biological and clinical relevance in gastric cancer, particularly in relation to angiogenesis, HER2 overexpression, lymph node dissemination, and poor prognosis.

An additional bioinformatic analysis was performed using the miRDB database (https://mirdb.org/), an online resource for microRNA target prediction. Predicted target genes of miR-27b-5p, miR-128-3p, miR-145-5p, and miR-552-3p were identified and subsequently evaluated for their potential involvement in HER2-associated signaling pathways.

### 4.4. Statistical Analysis

All analyses were performed in R version 4.3.2. No data imputation was performed, and all analyses were based on available cases, with complete-case analysis applied separately for each comparison or regression model. Continuous clinicopathological variables were summarized as mean and standard deviation, and categorical variables as counts and percentages.

For descriptive comparisons across clinicopathological subgroups, the microRNAs were analyzed on the log2 scale and summarized as median [Q1–Q3] with group-specific sample sizes. Normality was assessed with the Shapiro-Wilk test. For two-group comparisons, the unpaired t-test was used when both groups were approximately normally distributed and the Wilcoxon rank-sum test otherwise. Comparisons across more than two groups were performed with the Kruskal-Wallis test. Correlations between microRNA expression and quantitative FISH-derived variables, namely *CEP17* signals per nucleus, *HER2* signals per nucleus, and the *HER2*/*CEP17* ratio, were assessed on the raw expression scale. Pearson correlation was used when both variables had Shapiro-Wilk *p* > 0.05; otherwise, Spearman rank correlation was applied.

Overall survival was assessed in three complementary ways. Kaplan-Meier curves were generated after dichotomizing microRNA expression at the median and were compared with the log-rank test. Six perioperative deaths were excluded from the time-to-event risk sets. Cox proportional hazards regression was used for time-to-event analysis, and the proportional hazards assumption was checked with Schoenfeld residuals. Separate logistic regression models were fitted for survival status at 1, 2, 3, 4, and 5 years.

Logistic regression was also used for HER2-related outcomes, including IHC 3+ versus 0–2, IHC 2+/3+ versus 0/1, FISH amplification versus no amplification with equivocal cases excluded, composite HER2-positive versus HER2-negative status, and *CEP17* copy number increase versus no increase. For the regression analyses presented in the main tables, microRNA expression was modeled as a 1-standard-deviation increase in log2-transformed expression. Results are presented for unadjusted and fully adjusted models; the fully adjusted model included pT, pN, M, sex, and age. Odds ratios and hazard ratios are reported with 95% confidence intervals derived from confint() on the fitted models. For logistic and Cox regression, *p* values were obtained from likelihood ratio tests. All tests were two-sided, and *p* < 0.05 was considered statistically significant. All analyses were considered exploratory. Because the study evaluated a limited number of biologically related candidate microRNAs across several correlated outcomes in a relatively small cohort, no formal adjustment for multiple testing was applied. Accordingly, the reported *p* values should be interpreted as descriptive measures of the strength of association and used primarily to rank signals and support hypothesis generation.

## 5. Conclusions

This study provides new insights that selected microRNAs may be associated with HER2-related molecular alterations in gastric cancer. These findings extend current knowledge on HER2 regulation and may contribute to the development of more precise molecular stratification and targeted therapeutic strategies. However, our outcomes should be interpreted as exploratory and further studies are warranted to validate these findings and to better elucidate the underlying molecular mechanisms. A deeper understanding of the role of microRNAs in HER2-driven signaling pathways may provide a promising avenue for the development of novel microRNA-based therapeutic strategies.

## Figures and Tables

**Table 1:** Histopathological data and 5-year survival rate in the studied group.

Pathoclinical Feature	n = 115
pTNM	
I	18 (15.7%)
II	33 (28.7%)
III	59 (51.3%)
IV	5 (4.3%)
N+	85 (73.9%)
Mean (median) number of metastatic lymph nodes	6.1 (3)
Mean (median) number of resected lymph nodes	21.9 (20)
Lauren histopathological type	
1 Intestinal	56 (48.7%)
2 Diffuse	33 (28.7%)
3 Mixed	26 (22.6%)
HER2 IHC	
0	56 (48.7%)
1+	24 (20.9%)
2+	20 (17.4%)
3+	15 (13.0%)
*HER2* FISH	
No amlification	91 (79.1%)
Amplification	14 (12.2%)
Equivocal	10 (8.7%)
HER2 status	
negative	94 (81.7%)
positive	17 (14.8%)
Equivocal	4 (3.5%)
*CEP17* CNI	
Yes	27 (23.5%)
No	88 (76.5%)
Mean (median) number of *HER2* signals	3.7 (2.6)
Mean (median) number of *CEP 17* signals	2.6 (2.3)
Mean (median) *HER2*/*CEP17* ratio	1.37 (1.1)
5-year survival	40.0%

**Table 2:** Logistic regression of microRNA expression (per 1 SD of log2-transformed expression) and HER2-related outcomes. *p* values were obtained from likelihood ratio tests. Fully adjusted models included pT, pN, M, sex, and age. Note. Dashes indicate models that could not be estimated because of complete or quasi-complete separation. Asterisk indicates statistically significant associations (*p* < 0.05).

miRNA	Outcome	OR (Unadjusted)	95% CI (Unadjusted)	*p* (Unadjusted)	OR (Fully Adjusted)	95% CI (Fully Adjusted)	*p* (Fully Adjusted)
hsa-miR-21-5p	IHC 3+	0.909	0.54–1.59	0.7288	0.920	0.49–1.79	0.7999
	FISH amplification	0.913	0.52–1.65	0.7537	0.825	0.42–1.66	0.5824
	HER2 status	0.982	0.59–1.71	0.947	1.049	0.56–2.04	0.8827
	CEP17 CNI	0.959	0.62–1.50	0.8526	0.761	0.46–1.27	0.2904
hsa-miR-27b-5p	IHC 3+	1.096	0.52–2.26	0.8067	-	-	-
	FISH amplification	1.234	0.58–2.75	0.588	1.180	0.54–2.77	0.6838
	HER2 status	1.165	0.55–2.59	0.6932	-	-	-
	CEP17 CNI	1.858	1.06–3.53	0.0293 *	1.953	1.10–3.78	0.0215 *
hsa-miR-128-3p	IHC 3+	1.363	0.79–2.30	0.2556	1.467	0.80–2.71	0.214
	FISH amplification	1.096	0.60–1.91	0.7561	1.103	0.57–2.10	0.766
	HER2 status	1.249	0.74–2.05	0.3896	1.407	0.78–2.57	0.2527
	CEP17 CNI	1.109	0.71–1.70	0.6391	1.038	0.63–1.66	0.8772
hsa-miR-142-3p	IHC 3+	0.835	0.46–1.47	0.5358	0.823	0.42–1.55	0.5499
	FISH amplification	0.856	0.45–1.56	0.6153	0.783	0.38–1.52	0.4752
	HER2 status	0.915	0.52–1.58	0.7517	0.933	0.50–1.72	0.8241
	CEP17 CNI	0.820	0.51–1.30	0.4001	0.676	0.39–1.13	0.1359
hsa-miR-145-5p	IHC 3+	1.860	1.05–3.62	0.0329 *	2.159	1.06–5.18	0.0344 *
	FISH amplification	1.263	0.72–2.29	0.4195	1.306	0.66–2.72	0.4442
	HER2 status	1.597	0.94–2.90	0.0873	1.651	0.85–3.59	0.144
	CEP17 CNI	0.811	0.52–1.25	0.3434	0.946	0.56–1.59	0.8332
hsa-miR-187-3p	IHC 3+	0.870	0.47–1.67	0.6649	0.755	0.33–1.69	0.4873
	FISH amplification	0.735	0.39–1.38	0.3304	0.601	0.25–1.37	0.2264
	HER2 status	0.740	0.41–1.33	0.3111	0.556	0.24–1.21	0.1393
	CEP17 CNI	1.035	0.61–1.79	0.8984	1.006	0.56–1.85	0.9854
hsa-miR-196a-5p	IHC 3+	1.177	0.63–2.41	0.6258	1.142	0.55–2.55	0.7271
	FISH amplification	1.528	0.77–3.44	0.2385	1.514	0.69–3.79	0.3105
	HER2 status	1.256	0.69–2.50	0.4731	1.251	0.62–2.76	0.5443
	CEP17 CNI	1.253	0.73–2.30	0.4257	1.106	0.62–2.06	0.7343
hsa-miR-375	IHC 3+	1.054	0.60–1.87	0.8545	1.079	0.57–2.08	0.8148
	FISH amplification	0.914	0.51–1.64	0.7632	0.945	0.48–1.84	0.8653
	HER2 status	0.981	0.57–1.68	0.9443	0.989	0.53–1.85	0.9713
	CEP17 CNI	1.068	0.68–1.68	0.7737	1.165	0.73–1.88	0.5177
hsa-miR-382-5p	IHC 3+	1.492	0.87–2.58	0.1453	1.616	0.92–2.89	0.0934
	FISH amplification	1.350	0.77–2.37	0.294	1.479	0.82–2.70	0.1882
	HER2 status	1.363	0.81–2.30	0.2422	1.541	0.89–2.71	0.1206
	CEP17 CNI	0.915	0.58–1.42	0.6945	0.864	0.53–1.38	0.5429
hsa-miR-552-3p	IHC 3+	1.272	0.63–2.51	0.4884	1.311	0.58–2.93	0.5048
	FISH amplification	1.637	0.84–3.30	0.1457	2.208	0.95–5.68	0.0654
	HER2 status	1.427	0.76–2.70	0.2622	1.545	0.74–3.33	0.2478
	CEP17 CNI	1.360	0.81–2.33	0.2456	1.379	0.79–2.47	0.2583
hsa-miR-708-3p	IHC 3+	1.289	0.66–2.42	0.4374	1.641	0.80–3.65	0.1772
	FISH amplification	1.159	0.60–2.13	0.642	1.662	0.79–3.69	0.1798
	HER2 status	1.143	0.61–2.06	0.6633	1.518	0.77–3.15	0.2287
	CEP17 CNI	1.315	0.79–2.21	0.2891	1.434	0.80–2.68	0.225

**Table 3:** Correlations between miRNA expression and FISH-derived parameters. Pearson correlation was used when both variables were approximately normally distributed; otherwise, Spearman rank correlation was used.

	Number of *HER2* Signals per Nucleus		Number of *CEP17* Signals per Nucleus		Ratio	
	r	*p*	r	*p*	r	*p*
hsa-miR-21-5p	0.056	0.5519	0.06	0.5278	0.05	0.5959
hsa-miR-27b-5p	0.15	0.1987	0.106	0.3646	0.197	0.0905
hsa-miR-128-3p	0.234	0.0121	0.217	0.0204	0.191	0.0417
hsa-miR-142-3p	−0.081	0.4002	−0.067	0.4881	−0.036	0.7103
hsa-miR-145-5p	−0.157	0.0937	−0.225	0.0156	−0.08	0.3977
hsa-miR-187-3p	0.096	0.3869	0.105	0.3416	−0.026	0.8173
hsa-miR-196a-5p	0.107	0.3243	0.137	0.205	0.036	0.7427
hsa-miR-375	0.158	0.0957	0.117	0.2182	0.149	0.1162
hsa-miR-382-5p	0.074	0.446	0.086	0.3732	0.063	0.5162
hsa-miR-552-3p	0.281	0.0097	0.346	0.0013	0.098	0.3729
hsa-miR-708-3p	0.122	0.295	0.122	0.299	0.116	0.3213

**Table 4:** miRNA expression across clinicopathological features. Values are medians [Q1–Q3] of log2-transformed expression. Asterisks indicate statistically significant associations (* *p* < 0.05, ** *p* < 0.01, *** *p* < 0.001).

Feature	Group	hsa-miR-27b-5p (n = 74)	hsa-miR-128-3p (n = 114)	hsa-miR-145-5p (n = 115)	hsa-miR-552-3p (n = 84)
T	pT1–2 (n = 28)	−8.4 [−9.201 to −8.001] (n = 19)	−5.984 [−6.596 to −4.801] (n = 27)	4.212 [3.678 to 4.635] (n = 28)	−9.692 [−11.565 to −8.752] (n = 20)
	pT3–4 (n = 87)	−8.615 [−9.15 to −7.72] (n = 55)	−5.093 [−6.34 to −3.796] (n = 87)	3.102 [2.331 to 3.797] (n = 87)	−9.733 [−10.86 to −8.22] (n = 64)
	*p*	0.7159	0.0658	<0.0001 ***	0.5084
pN	N0 (n = 30)	−8.329 [−9.047 to −7.77] (n = 21)	−5.895 [−6.399 to −4.652] (n = 30)	3.758 [2.774 to 4.541] (n = 30)	−9.518 [−10.484 to −8.012] (n = 22)
	N+ (n = 85)	−8.627 [−9.27 to −7.806] (n = 53)	−5.139 [−6.325 to −3.765] (n = 84)	3.272 [2.458 to 3.888] (n = 85)	−9.824 [−11.07 to −8.377] (n = 62)
	*p*	0.4108	0.1209	0.0804	0.44
M	M0 (n = 110)	−8.601 [−9.184 to −7.769] (n = 72)	−5.493 [−6.381 to −3.861] (n = 109)	3.433 [2.559 to 4.096] (n = 110)	−9.727 [−11.061 to −8.343] (n = 81)
	M1 (n = 5)	−8.583 [−8.677 to −8.489] (n = 2)	−5.054 [−6.118 to −4.11] (n = 5)	3.575 [3.272 to 3.879] (n = 5)	−10.747 [−10.747 to −8.49] (n = 3)
	*p*		0.6479	0.6808	0.9041
pTNM stage	I–II (n = 51)	−8.504 [−9.12 to −7.951] (n = 34)	−5.917 [−6.516 to −4.539] (n = 50)	3.658 [2.615 to 4.427] (n = 51)	−9.75 [−11.42 to −8.312] (n = 38)
	III–IV (n = 64)	−8.618 [−9.186 to −7.74] (n = 40)	−4.978 [−6.141 to −3.495] (n = 64)	3.206 [2.529 to 3.843] (n = 64)	−9.733 [−10.768 to −8.377] (n = 46)
	*p*	0.7981	0.0207 *	0.0829	0.4597
Mucinous	No (n = 87)	−8.612 [−9.263 to −7.714] (n = 52)	−5.168 [−6.198 to −3.523] (n = 86)	3.18 [2.415 to 3.885] (n = 87)	−9.658 [−10.893 to −8.012] (n = 62)
	Yes (n = 28)	−8.398 [−9.11 to −7.844] (n = 22)	−5.998 [−6.53 to −4.914] (n = 28)	3.883 [3.05 to 4.489] (n = 28)	−9.914 [−10.983 to −8.984] (n = 22)
	*p*	0.9811	0.034 *	0.0147 *	0.4333
Tumor location	Cardia (n = 41)	−8.627 [−9.206 to −7.966] (n = 27)	−5.417 [−6.211 to −4.195] (n = 41)	3.023 [2.392 to 3.645] (n = 41)	−9.818 [−10.404 to −8.233] (n = 32)
	Other (n = 74)	−8.593 [−9.15 to −7.72] (n = 47)	−5.594 [−6.506 to −3.79] (n = 73)	3.671 [2.774 to 4.225] (n = 74)	−9.658 [−11.287 to −8.332] (n = 52)
	*p*	0.5262	0.7209	0.0142 *	0.4229
Lauren	Intestinal (n = 56)	−8.691 [−9.27 to −7.809] (n = 33)	−4.908 [−6.209 to −3.662] (n = 55)	2.953 [1.918 to 3.7] (n = 56)	−8.934 [−10.063 to −7.54] (n = 42)
	Diffuse (n = 33)	−8.65 [−9.201 to −8.075] (n = 27)	−6.078 [−6.951 to −5.054] (n = 33)	3.879 [3.568 to 4.167] (n = 33)	−11.061 [−12.218 to −9.593] (n = 27)
	Mixed (n = 26)	−7.946 [−8.622 to −7.766] (n = 14)	−5.064 [−6.084 to −3.718] (n = 26)	3.332 [2.769 to 4.081] (n = 26)	−10.147 [−10.975 to −8.749] (n = 15)
	*p*	0.4143	0.0306 *	0.0035 **	0.0008 ***

**Table 5:** Logistic regression of miRNA expression (per 1 SD of log2-transformed expression) and survival status at 1, 2, 3, 4, and 5 years. *p* values were obtained from likelihood ratio tests. Fully adjusted models included pT, pN, M, sex, and age. Asterisks indicate statistically significant associations (* *p* < 0.05, ** *p* < 0.01).

miRNA	Outcome	OR (Unadjusted)	95% CI (Unadjusted)	*p* (Unadjusted)	OR (Fully Adjusted)	95% CI (Fully Adjusted)	*p* (Fully Adjusted)
hsa-miR-27b-5p	OS at 1 year	0.497	0.25–0.91	0.0232 *	0.418	0.18–0.82	0.0093 **
	OS at 2 years	0.626	0.36–1.03	0.0658	0.526	0.27–0.92	0.0237 *
	OS at 3 years	0.715	0.43–1.14	0.1634	0.526	0.27–0.95	0.0323 *
	OS at 4 years	0.849	0.52–1.35	0.4898	0.733	0.41–1.27	0.2685
	OS at 5 years	0.954	0.59–1.53	0.8426	0.879	0.50–1.52	0.6446
hsa-miR-128-3p	OS at 1 year	1.875	1.09–3.55	0.022 *	1.912	1.06–3.81	0.0311 *
	OS at 2 years	1.140	0.78–1.70	0.5038	1.289	0.84–2.04	0.2527
	OS at 3 years	1.045	0.72–1.52	0.8166	1.291	0.82–2.05	0.266
	OS at 4 years	1.169	0.81–1.71	0.4111	1.510	0.96–2.43	0.0732
	OS at 5 years	1.264	0.87–1.86	0.222	1.648	1.04–2.68	0.0316 *
hsa-miR-145-5p	OS at 1 year	1.212	0.76–1.95	0.4193	1.208	0.68–2.19	0.5225
	OS at 2 years	1.333	0.91–2.00	0.1441	1.209	0.75–1.97	0.4363
	OS at 3 years	1.713	1.15–2.66	0.0071 **	1.374	0.83–2.34	0.215
	OS at 4 years	1.708	1.14–2.66	0.0079 **	1.287	0.79–2.17	0.3167
	OS at 5 years	1.557	1.05–2.41	0.0274 *	1.172	0.72–1.96	0.5269
hsa-miR-552-3p	OS at 1 year	1.167	0.69–2.04	0.5692	1.019	0.56–1.91	0.9524
	OS at 2 years	1.286	0.82–2.08	0.2786	1.255	0.76–2.13	0.3774
	OS at 3 years	1.165	0.76–1.82	0.489	1.226	0.73–2.09	0.4412
	OS at 4 years	1.222	0.79–1.93	0.3709	1.295	0.78–2.18	0.3151
	OS at 5 years	1.276	0.82–2.03	0.2824	1.288	0.78–2.17	0.3245

**Table 6:** Cox regression of miRNA expression (per 1 SD of log2-transformed expression) and overall survival. *p* values were obtained from likelihood ratio tests. Fully adjusted models included pT, pN, M, sex, and age. Asterisk indicates statistically significant associations (* *p* < 0.05).

miRNA	Outcome	HR (Unadjusted)	95% CI (Unadjusted)	*p* (Unadjusted)	HR (Fully Adjusted)	95% CI (Fully Adjusted)	*p* (Fully Adjusted)
hsa-miR-27b-5p	Overall survival	1.12	0.83–1.49	0.4593	1.40	1.03–1.90	0.0338 *
hsa-miR-128-3p	Overall survival	0.87	0.70–1.07	0.1826	0.79	0.63–0.99	0.0439 *
hsa-miR-145-5p	Overall survival	0.85	0.70–1.05	0.1269	1.04	0.80–1.35	0.7605
hsa-miR-552-3p	Overall survival	0.92	0.74–1.15	0.4681	0.95	0.75–1.19	0.6408

**Table 7:** Association between the presence or absence of *CEP17* CNI and survival.

Probability of Survival	1-Year	2-Year	3-Year	4-Year	5-Year
*CEP17* CNI Yes n = 27	74.1%	48.1%	33.3%	29.6%	25.9%
*CEP17* CNI No n = 88	83.0%	67.0%	53.4%	48.9%	44.3%
*p*	0.40	0.11	0.08	0.12	0.12

## Data Availability

The raw data supporting the conclusions of this article will be made available by the authors on request.

## Abbreviations

The following abbreviations are used in this manuscript:

HER2	Human Epidermal Growth Factor receptor 2
CNI	Copy number increase
IHC	Immunohistochemistry
FISH	Fluorescence in situ hybridization
CEP17	Centromere enumeration probe for chromosome 17
NGS	Next-generation sequencing
EMT	Epithelial-mesenchymal transition
